# Assessment of nurses' knowledge, attitude and implementation of skin‐to‐skin care within the perinatal setting in Saudi Arabia: Survey study

**DOI:** 10.1002/nop2.1464

**Published:** 2022-11-10

**Authors:** Abbas Al Mutair, Wedad Almutairi, Faiza Aljarameez, Elbert Kay, Rhea Rabanal, Katrina Abellar, Aireen Napaod, Abeer Hawsawi, Chandni Saha, Gasmelseed Y. Ahmed

**Affiliations:** ^1^ Research Center Almoosa Specialist Hospital Alhassa Saudi Arabia; ^2^ School of Nursing University of Wollongong Wollongong New South Wales Australia; ^3^ Medical Surgical Nursing Department Princess Nourah Bint Abdulrhamn University Riyadh Saudi Arabia; ^4^ Nursing Department Almoosa College of Health Sciences al‐Mubarraz Saudi Arabia; ^5^ Department of Maternity and Child Nursing Faculty of Nursing King Abdulaziz University Jeddah Saudi Arabia; ^6^ King Saud Bin Abdulziz University for Health Sciences Alhasa Saudi Arabia; ^7^ King Abdullah International Medical Research Center Alhasa Saudi Arabia; ^8^ Ministry of National Guard‐Health Affairs Alhasa Saudi Arabia; ^9^ Department of Maternity and Child Nursing Faculty of Nursing King Saud University Riyadh Saudi Arabia; ^10^ College of medicine and health sciences Almangil University Almangil Sudan

**Keywords:** neonate, perinatal care, Skin‐to‐skin care (Kangaroo mother care)

## Abstract

**Aim:**

To evaluate knowledge, attitude and implementation of Skin‐to‐Skin Care (SSC) among nurses and to assess the implementation of SSC in the perinatal setting.

**Design:**

A cross‐sectional study design was implemented utilizing survey distributed among critical care paediatric and neonatal nurses.

**Methods:**

The data collection form was pre‐structured validated tool consisted of two main sections; socio‐demographic characteristics and Likert scale of 20 items covering four main domains in; knowledge, attitude, education and implementation of SSC.

**Results:**

The vast majority of the sample 91 (98%) were females with mean age and mean years of experience 33.5 ± 6.5 and 9.7 ± 6.5 years respectively. Almost half of them 45 (48.4%) work in obstetric and labor and delivery units. Correlation coefficient revealed a significant association between the total educational years of nursing degree and SSC. The results showed nurses with bachelor or master significantly more knowledgeable and skilled in implementing SSC compared to others.

## 1 BACKGROUND

Kangaroo Mother Care (KMC), which is a skin‐to‐skin contact (SSC) of baby with the mother is recommended not only for preterm but also for full term baby as it is a very robust and cost‐effective way of promoting the health and wellbeing of the baby (World Health Organization [WHO], 2003). KMC was originated from the fact that it employs similar method of marsupial caregiving (Al‐Shehri & Binmanee, 2021). KMC is practiced right from birth and continues afterwards at home. The baby is placed on the chest between the breast below the clothes for a minimum of 1 hr just after birth to initiate breast feeding, regulate the baby's body temperature and cardiovascular function. Studies have shown that this helps the baby to cope with pain, reduces apnoea during a quiet sleep and improves blood oxygenation (Al‐Shehri & Binmanee, 2021; Shattnawi et al., 2019). SSC is usually performed in Neonatal Intensive Care Unit (NICU) where the bond of the parents and the newborn infant is primarily established through innovative nursing practices. The earliest touch from the parents is vital to the overall well‐being of the infant. Moreover, the ability of the infant to have significant connection with the outside world was honed with the first moments that the caregivers held the infant as they deliver the needed care. Truly, nurses portray a vital role in order for SSC to actively take place especially that the mother‐infant interaction occurs and falls within the scope of nursing practice. According to Charpak and Gabriel Ruiz (2011), the Kangaroo care is not seen in advanced NICU environment but expert groups and 7th KMC international workshop emphasized the importance of active and quality KMC (prolonged skin to skin contact) even in NICU with high‐end facilities (Nyqvist et al., 2010).

Kangaroo Mother Care was first introduced in Bogota, Colombia in 1978 by Edgar Rey and Martinez as an alternative method to maintain body temperature of baby using skin to skin contact rather than an incubator (Albishry et al., 2018). It has been found that 53% of mortality is associated with neonatal morbidity and KMC can reduce mortality rate by 36% (Al‐Shehri & Binmanee, 2021; Conde‐Agudelo & Díaz‐Rossello, 2016). This was highlighted in the WHO report (2003) where WHO recommends KMC for low birth baby and premature stable baby weighing 2000 grams or less as it helps to reduce mortality rate (WHO, 2015; World Health Organisation, 2003). While KMC is beneficial to baby, it can also benefit mothers as it helps create a stronger mother–baby bond, as well as it helps mothers to cope with anxiety and postpartum depression as SSC triggers release of oxytocin (Badr & Zauszniewski, 2017). It has also been reported to lower the incidence of primary postpartum haemorrhage (Abdulghani, Amir, & Edvardsson, 2020; Abdulghani, Edvardsson, & Amir, 2020).

The practice is new in Saudi Arabia. In one study carried out in the Kingdom to observe 22 healthy mother–infant dyads and collect information on duration of SSC, any disturbance and breastfeeding practices using the Birthing Room Audit Tool developed by Cantrill and colleagues in 2014 (Abdulghani, Amir, & Edvardsson, 2020; Cantrill et al., 2014) it was found that although 18 mothers could only hold their baby for 2 days it had a direct SSC while 16 of them had the baby placed on their chest or abdomen with a barrier only for an average of 4 min (Abdulghani, Edvardsson, & Amir, 2020). Various barriers to SSC were identified such as SSC being interrupted several times in the first hour by clinicians completing the assessment or nurses giving vitamin K injections or weighing or dressing the baby. This is one of the most important steps of KMC that is not being practiced properly and therefore does not provide the infants with all the benefits associated with SSC (Abdulghani, Edvardsson, & Amir, 2020).

Several studies about knowledge, attitude and practice have highlighted the need to engage all stakeholders to develop clear guidelines about KMC and to facilitate it through training and increasing the numbers of nurses to be able to practice KMC (Abdulghani, Amir, & Edvardsson, 2020; Abdulghani, Edvardsson, & Amir, 2020; Albishry et al., 2018; Al‐Shehri & Binmanee, 2021). A study carried out in Jordan showed very good knowledge and attitudes towards KMC but the practice as indicated by the authors was affected by the lack of training, clear guidelines and policies and lack of organizational support (Shattnawi et al., 2019). Similar findings were published by Al‐Shehri and Binmanee (2021) where the knowledge among nurses about KMC was very good but the practice was impacted by various barriers such as a fear to practice KMC especially if the baby is intubated, family resistance and parent's discomfort. It was found out that 92.8% of the nurses would encourage parents about KMC and around 90% provided information to parent but expert advice was given to only 67.5% of women. None of the studies reported touched on the practice after discharge and also less is known about the knowledge, attitude and practice of KMC among mothers. Alnajjar (2012) reported resistance towards the practice from husband based on strong religious beliefs as well as a lack of privacy in public hospitals. After an extensive literature search it was observed that though assessing nurses' knowledge and attitude towards SSC is critical to mother–infant health, there were no related studies assessing the perception of nurses’ in the Al‐ahsa region of Saudi Arabia. The studies available are from the city of Riyadh (Al‐Shehri & Binmanee, 2021) and Jeddah (Abdulghani, Edvardsson, & Amir, 2020; Almutairi, 2022) however, the absence of studies from Al‐ahsa governorate needs significant notice as it holds largest population in the eastern region of Saudi Arabia. Therefore, finding it imperative the present was conducted to discern the nurses’ knowledge, skills, beliefs and perception.

### 1.1 Purpose of the study

The study was conducted to identify nurses' knowledge, skills and attitude regarding SSC and to assess their belief and perceptions towards the practice of SSC.

## 2 METHODS

A survey design was used to conduct the study among all nurses in the maternal related units (labor and delivery, NICU and Postpartum) regarding SSC in neonates cared for within the perinatal setting. A convenience sampling technique was used in the study to ask every nurse available in the related units to participate in the study. The inclusion criteria include all nurses who were working in the labor and delivery, nicu, postpartum. All the nurses who did not work or deal with mothers and newborns were excluded in the present study. An ethical clearance to conduct the study was obtained from Institutional Review Board “REDACTED”.

The survey utilized in the present study was first introduced by S. Ludington‐Hoe, and consists of 21 questions related to nurses' knowledge, attitudes and beliefs regarding SSC. Perceptions of SSC guidelines and protocols are included among the survey questions. Items were scored by participants with a five‐point Likert‐type scale ranging from Strongly Disagree to Strongly Agree. Content validity was supported by a cross‐disciplinary review of the literature conducted using CINAHL, PsycInfo, PubMed and the Cochrane Library, as well as Scopus databases. Construct validity was established using alpha reliability of 0.79–0.90. The survey contained four constructs: SSC implementation, SSC knowledge, SSC attitudes and beliefs, and SSC education and training. Data were also collected for six additional demographic questions to identify participants' gender, age, nationality, education level, years of experience, and working area.

### 2.1 Data analysis

Data were entered into excel sheet for cleaning, accuracy and managing missing values. Upon data completion and verification, data were transported from excel to the statistical software; statistical package for social science (SPSS) V25 for analysis. Both descriptive and inferential statistics were conducted, *p*‐value of ≤.05 were accepted as significance level for all statistical tests. Continuous variables including participant's age and years of experience for nurses were expressed and reported as mean ± standard deviations. Categorical variables including gender, nationality and nurses' qualification were presented as counts and proportions (%). Univariate analysis for comparison of different categorical variables against continues outcomes variables, reporting paired *t*‐test test were applied. Multivariate analysis were also conducted to assess the association between categorical and continuous demographic characteristics. The correlational coefficient was also used to study the association between the scale numerical variables.

## 3 RESULTS

Data on 93 nurses work in intensive care units of Almoosa Specialist Hospital (ASH), Al‐Ahsa, Saudi Arabia were extracted and analysed applying SPSS version 25, where both descriptive and inferential analysis were performed on the socio‐demographic characteristics and the four dimensions of the SSC scale's items. The majority of the nurses 91 (98%) were females with mean age and mean years of experience of 33.5 ± 6.5 and 9.7 ± 6.5 years respectively, almost half of them 45 (48.4%) works in obstetric and labor and delivery units, Table 1.

**TABLE 1:** Socio‐demographic characteristics (*n* = 93)

Characteristics	*N* (%)
Gender	
Males	02 (02.2%)
Females	91 (97.8%)
Nationality	
Saudi	05 (05.4%)
Non‐Saudi	88 (94.6%)
Nursing qualification	
Diploma	24 (25.8%)
Bachelor	67 (72.0%)
Master	02 (02.2%)
Working area	
OB ward	10 (10.8%)
Nursery	17 (18.3%)
NICU	31 (33.3%)
Labor and delivery	35 (37.6%)
Age	33.5 ± 6.5 years
Years of Nursing Experience	9.7 ± 6.5
Years of Experience in your current area	6.1 ± 5.7

The statistical analysis results revealed that, nurses graduated from educational programs of 4 years and more that offer a bachelor or a master degrees in nursing were more confident than others in their knowledge, attitude and implementation of SSC. They have reported positive responses (strongly agree and agree), 78% of them were positive when interviewed about their confidence in ability to interpret infant responses during SSC, 82% agree about feeling confident with skills in recognizing and assessing the physiologic and behavioural responses of infants during SSC and 83% were feeling confident in skills to safely facilitate skin‐to‐skin holding with neonates, Table 2. The results from Table 3 highlight the nurses' participated perceived having reasonable knowledge and attitude levels about SSC.

**TABLE 2:** Proportions of the items of the four dimensions of SSC (*n* = 93)

Dimensions' question	Strongly agree (%)	Agree (%)	Neutral (%)	Disagree (%)	Strongly disagree (%)
Knowledge					
I am confident in my ability to interpret infant responses during SSC	40 (43.0)	38 (40.9)	08 (08.6)	00 (00.0)	07 (07.5)
Lack of SSC in the neonatal period has long term adverse effects.	26 (28.0)	47 (50.4)	18 (19.4)	01 (01.1)	01 (01.1)
SSC can reduce the risk of impaired brain development in neonates	19 (20.4)	46 (49.5)	19 (20.4)	05 (05.4)	04 (04.3)
I feel confident with my skills in recognizing & assessing thephysiologic/behavioural responses of infants during SSC.	33 (35.5)	49 (52.7)	06 (06.5)	03 (03.2)	02 (02.2)
SSC changes brain growth in the neonate.	16 (17.2)	56 (60.2)	16 (17.2)	02 (02.2)	03 (03.2)
Attitudes and believes					
Discomfort from minor procedures, such as gavage tube placement & oral suctioning can be minimized with SSC	09 (09.7)	48 (51.6)	25 (26.9)	10 (10.8)	01 (01.1)
It is the responsibility of nurses to be an advocate for skin‐to‐skinholding for neonates in their care.	47 (50.5)	35 (37.6)	07 (07.5)	01 (01.1)	03 (03.2)
SSC is effective in reducing risks associated with physical separation of the neonate and their mother.	37 (39.8)	43 (46.2)	08 (08.6)	03 (03.2)	02 (02.2)03 (03.2)
I feel confident in my skills to safely facilitate skin‐to‐skin holding with neonates.	40 (43.0)	43 (46.2)	06 (06.5)	01 (01.1)	
Education					
My unit provides continuing education regarding SSC.	22 (23.7)	40 (43.0)	19 (20.4)	08 (8.6)	04 (04.3)
I am aware of SSC guidelines/protocols in my unit.	21 (22.6)	45 (48.4)	18 (19.4)	05 (05.4)	04 (04.3)
My unit regularly uses an assessment tool for SSC responses.	12 (12.9)	43 (46.2)	18 (19.4)	16 (17.2)	04 (04.3)
The way we measure responses to SSC on my unit is an accuratemeasurement of infant responses.	12 (12.9)	49 (52.7)	16 (17.2)	12 (12.9)	04 (04.3)
I have attended an educational course/conference/lecture that hasincluded SSC within the last 5 years.	07 (07.5)	33 (35.5)	25 (26.9)	21 (22.6)	07 (07.5)
Implementation					
I feel that the provision of SSC on my unit is well managed.	13 (14.0)	51 (54.8)	16 (17.2)	09 (09.7)	04 (04.3)
My unit uses skin to skin holding regularly.	16 (17.2)	47 (50.5)	21 (22.6)	06 (06.5)	03 (03.2)
The health care providers in my unit practice adequate SSC witheligible neonates.	13 (14.0)	58 (62.4)	16 (17.2)	04 (04.3)	02 (02.2)
Physicians are willing to use new evidence‐based applications of SSCon my unit.	07 (07.5)	50 (53.8)	28 (30.1)	06 (06.5)	02 (02.2)
I have received adequate education or training regarding SSC when Iwas oriented to my unit.	06 (06.5)	47 (50.5)	20 (21.5)	17 (18.3)	03 (03.2)
The SSC guidelines/protocols are clear, comprehensive, and based oncurrent research.	13 (14.0)	51 (54.8)	19 (20.4)	06 (06.5)	04 (04.3)

**TABLE 3:** Mean and SD for the each diminutions (*n* = 93)

Different sections of the dimensions	Mean ± SD
SSC knowledge	19.9 **±** 3.4
Attitude and believes	16.3 **±** 2.9
Education	17.7 **±** 4.3
Implementation	21.8 **±** 4.6
Total dimensions	75.8 **±** 12.2

Correlation coefficient test showed younger and recently fresh graduated nurses as having non‐significant, but more knowledge, attitude and implementation skills of SSC compared to old graduates, Table 4. However, there was a significant association between the total educational years of nursing degree and SSC (*p*‐value .02), Table 5. Hence nurses with master's and bachelor's degrees were shown to be significantly more knowledgeable and skilled in implementing SSC compared to those with the diploma, reflecting the role of the educational of academic curriculum and the nursing clinical training programs' significant influence on knowledge, attitude and implementation of SSC.

**TABLE 4:** Correlation between age and years of experience versus SSC score (*N* = 93)

Characteristics	Correlation coefficient	*p*‐Value
Correlation between age and SSC	−.02	.8
Correlation between experience and SSC	−.08	.9

**TABLE 5:** Association between educational degree and nursing SSC (*N* = 93)

Characteristics	Mean	*p*‐Value
Nursing qualification		.02
Diploma	14.7	
Bachelor and master	16.9	

## 4 DISCUSSION

Assessment of knowledge and attitudes is a major determinant of individual's behaviour. This study aimed to evaluate knowledge, attitude and implementation of SSC among nurses in the maternal related units labor and delivery, NICU and Postpartum working at tertiary private hospital, Saudi Arabia. The findings of this study indicated that most of the participants perceived having reasonable knowledge and attitude levels about SSC. This finding is congruent with Al‐Shehri and Binmanee (2021) study on NICU nurses in Riyadh, Saudi Arabia. Similarly, Shattnawi et al. (2019) found that NICU nurses in 12 Jordanian public hospitals had positive perceptions regarding the impact of SSC to maintain infant–mother relationships and quality of care. A possible explanation for this finding might be related to the proper staff education and regular assessment by the experts of nurses' knowledge and skills of SSC. In a study by Dalal et al. (2014), a significant association was reported between employing educational and training programs for nurses and their knowledge scores of SSC. Nonetheless, in order to ensure the quality and effectiveness of provided educational programs, personal perceptions and attitudes of nurses towards SSC should be given meticulous consideration. Moreover, to improve nurses’ perceptions and practice, assessing the confounding factors towards SSC must be considered. Globally, various research studies reported barriers to nurses’ engagement in SSC practice, including women post‐natal practice, reluctance and fear of parents, level of nurses’ experience, nurse–patient ratio, staff shortage, safety consideration, medical staff attitude, lack of management support (Deng et al., 2018; Flynn & Leahy‐Warren, 2010; Yue et al., 2020).

Another possible explanation of the reasonable knowledge and attitude about SSC is the fact that the participants of this study work in a tertiary level hecare facility. This type of hospital setting is expected to provide profound level of training and regular assessment of their healthcare workers to improve their skills and experience. Therefore, it is recommended that healthcare settings support examining perceptions of healthcare workers towards SSC in order to provide appropriate education and training programs to overcome the barriers to provide adequate SSC practice.

Nurses have a positive perception about the positive health outcomes of SSC. In fact, this serves as a passport to bring health care reforms in the care given for the mother and infant with relation to the SSC (Chia et al., 2006; Engler et al., 2002; Semenic et al., 2012; Wallin et al., 2005). Chia et al. (2006) conducted a study investigating nursing attitudes regarding the practice of SSC in the NICU where in there was a total of 34 nurses in Melbourne, Australia that accomplished a descriptive survey and four nurses gave an in‐depth interview. According to Chia et al. (2006) nurses were able to feel more compassion when they assist in the implementation of SSC with a hope also from them that the parents would also wish for en early SSC with the infant. Based on the observation of the nurses in the said study, the parents enhanced their bond with the infant. Also, infants who were undergoing SSC over a period of time tend not to cry a lot. Mothers in SSC had less anxiety and expressed satisfaction. Breastfeeding became more active resulting to generous milk production (Chia et al., 2006). Thus, nurses are encouraged to be involved in SSC educational activities (Chia et al., 2006; Semenic et al., 2012; Wallin et al., 2005).

Correlation analyses of the present study provided that nurses with a bachelor or a master degrees in nursing were more confident than others in their knowledge, attitude and implementation of SSC as compared to those having diploma degrees. This might be attributed to the sample characteristics in which almost two third of the participants hold a bachelor degree (*n =* 67, 72.0%). Another possible explanation is that nurses' knowledge, attitude and skills could be improved following continuing education and training sessions. Additionally, the findings of this study indicated (though non‐significant) that younger and recently fresh graduated nurses as well as experienced nurses reported better knowledge, attitude and implementation of SSC compared to old graduates. A possible explanation for this finding is that those with more experience must have encountered more difficulties and challenges in practice. For the recent nursing graduates, this might be attributed to the notion that this group still have the fresh knowledge and enthusiasm and look for opportunities to develop new skills and knowledge. Thus, it is recommended that nurses with lower educational degrees as well as fresh graduates receive continuing education and training programs (Almutairi & Ludington‐Hoe, 2016; Deng et al., 2018) to improve and maintain their knowledge and skills and develop more experience to overcome difficulties and barriers to practice SSC.

Based on the study entitled, “Neonatal Nurses' Perceptions of Supportive Factors and Barriers to the implementation of skin‐to‐skin care in extremely low birth weight (ELBW) infants ‐ A Qualitative Study” conducted by Suzie Lim on December 2017 that teaching approaches must be an important component in the staff orientation sessions. Moreover, doing bedside conferences is one of the teaching techniques during staff training especially in honing the knowledge, skills and attitude of the nurses in the implementation of SSC even to Extremely Low Birth Weight Infants. Importantly, the necessary skills and competencies were defined in the successful roll out and sustenance in the practice of SSC which are deeper knowledge, more experience and buoyancy on the evaluation of the nurses on the preparedness of the infant for the SSC. Nurses believed that informal education, actual bedside training and classes can facilitate their learning on SSC.

Evidence‐based practice should be the priority of the healthcare institutions especially among nurses who must uphold the highest standard of their profession considering they comprise the biggest workforce in every hospital (Institute of Medicine, 2010).

### 4.1 Limitations

There were certain limitations in this study. The study included a small homogenous sample size obtained from one tertiary hospital. Although the findings of this study provide better understanding of nurses' attitude and knowledge about SSC practices, sample characteristics, the cross‐sectional nature and the use of self‐reported measure could limit generalizability of the findings in this study. Therefore, it would be better to conduct studies with larger samples to compare SSC practices in different health care settings.

## 5 CONCLUSION

The findings of the present study support a reasonable knowledge regarding SSC and proper attitude and engagement levels of nurses working in perinatal areas. The study provided an insight into nurses' perceptions of SSC in a tertiary level hospital. In order to enhance SSC practice, considering providing educational and training programs of nurses is recommended. This could be achieved by involving all concerned stakeholders including health care providers, authority bodies and parents to establish clear evidence‐based SSC guidelines. Future studies could probably focus on barriers and facilitators of SSC practice in different hospital settings.

## AUTHOR CONTRIBUTIONS

AA: conception, proposal development, ethical approval, data recruitment, formal analysis and manuscript preparation. WA: conception, proposal development. FA: manuscript preparation. EK: manuscript refinement. RR: data recruitment. KA: data recruitment. AN: data recruitment. AH: manuscript preparation. CS: manuscript refinement. GA: formal analysis.

All authors have agreed on the final version and meet at least one of the following criteria [recommended by the ICMJE (http://www.icmje.org/recommendations/)]:

- substantial contributions to conception and design, acquisition of data or analysis and interpretation of data;
- drafting the article or revising it critically for important intellectual content.

## ETHICS STATEMENT

Participation in the study was voluntary and this was clearly indicated in the explanatory statement. It was also indicated that data confidentiality and privacy will be maintained. The collected data will be preserved for three years as per IRB regulations and then it will be discarded and shredded.
